# Modulatory effects of miracle fruit ethanolic extracts on glucose uptake through the insulin signaling pathway in C2C12 mouse myotubes cells

**DOI:** 10.1002/fsn3.935

**Published:** 2019-02-05

**Authors:** Yi‐Chun Han, Ju‐Yu Wu, Chin‐Kun Wang

**Affiliations:** ^1^ Department of Nutrition Chung Shan Medical University South District Taichung Taiwan, ROC

**Keywords:** antidiabetic effects, glucose uptake, insulin signal, miracle fruit, type 2 diabetes

## Abstract

Miracle fruit, *Synsepalum dulcificum*, is commonly known to be an alternative sweetener. It makes sour food taste sweet by affecting the tongue's taste receptors. It also shows beneficial health effects, such as antioxidant, anti‐inflammatory, and antihyperglycemic activities. This study was conducted to investigate the antidiabetic effects of miracle fruit flesh (MF) and seed (MS) ethanolic extracts and the underlying mechanisms. Differentiated C2C12 myotubes were treated with the MF or MS extract (1–1,000 μg/ml) or metformin (1 mM) in the presence or absence of insulin. Compared with metformin, the MF extract significantly increased the intake of 2‐(N‐(7‐nitrobenz‐2‐oxa‐1, 3‐diazol‐4‐yl) amino)‐2‐deoxyglucose (2‐NBDG). The MF extract also upregulated insulin receptor, phosphatidylinositol 3‐kinase, and glucose transporter 4 expressions. These results reveal the antidiabetic effects of miracle fruit.

## 1 INTRODUCTION

The incidence of diabetes is increasing annually, and by 2030, more than 366 million individuals are expected to be affected by diabetes; moreover, diabetes is expected to become the seventh leading cause of death (WHO, 2016; Wild, Roglic, Green, Sicree, & King, 2004). Diabetes mellitus (DM), particularly type 2 DM, is a chronic metabolic disorder with worldwide prevalence. It is characterized by a high blood glucose level (hyperglycemia), which leads to secondary complications in peripheral tissues. Type 2 DM (non‐insulin‐dependent DM) is the common type. More than 95% of patients with DM are type 2, which is characterized by insulin resistance (American Diabetes Association, 2012). Pharmacological agents, including sulfonylureas, thiazolidinedione, and metformin, have been used to treat type 2 DM. However, these agents have a limited dosage because of their undesirable side effects and their failure to significantly ameliorate diabetic complications (Rang, Dale, & Ritter, 1991). Recently, many types of fruits, vegetables, and medicinal plants and their products or phytochemicals have been reported to increase insulin secretion and sensitivity, thus leading to the emergence of renewed interest in alternative medicines and natural therapies to treat type 2 DM. Therefore, numerous studies have screened for novel materials or compounds that effectively elevate glucose metabolism without producing side effects.

*Synsepalum dulcificum*, commonly known as miracle fruit or red berry, is a tropical West African evergreen shrub (Daniell, 1985; Inglett & May, 1968). Miracle fruit is approximately 2−3 cm in length; it is oval shaped with red skin and contains a single seed covered by a thin layer of berry flesh, which has a slightly sweet flavor (Inglett & Chen, 2011). Miracle fruit has been used in medicine and in food as a low‐calorie, sugar‐free alternative sweetener; when low‐pH foods are consumed, the active compound of miracle fruit, miraculin, binds to the taste buds that recognize sweetness (Giroux & Henkin, 1974). Studies have shown that miracle fruit contains abundant antioxidant phytochemicals such as hydrophilic phenolic acids (Wang et al., 2011), ascorbic acid, lipophilic α‐tocopherol, and carotenoid (Du, Shen, Zhang, Prinyawiwatkul, & Xu, 2014). These phytochemicals may contribute to the antioxidant ability of miracle fruit and the diabetes, and cancers. Miracle fruit was also shown to improve insulin resistance induced by fructose (Fru)‐rich chow in rats (Chen, Liu, & Cheng, 2006).

The effects of miracle fruit on glucose metabolism and the underlying mechanism remain poorly understood. Therefore, this study was conducted to evaluate the effect of miracle fruit flesh (MF) and seed (MS) ethanolic extracts on glycation and glucose uptake and determine their underlying mechanism in the C2C12 muscle cell model.

## 2 MATERIALS AND METHODS

### 2.1 Chemical reagents

3‐[4, 5‐Dimethylthiazol‐2‐yl]‐2, 5‐diphenyltetrazolium bromide (MTT), and metformin, as well as all organic solvents, including 95% ethanol and dimethyl sulfoxide (DMSO), and the chemical reagents used for polyphenol, antioxidant, and antiglycation assays were purchased from Millipore Sigma (St. Louis, MO, USA). The commercial kits for the insulin receptor (IR) and glucose transporter 4 (GLUT4) were purchased from Biovision (San Francisco, CA, USA). Cell culture medium, insulin, and 2‐(n‐(7‐nitrobenz‐2‐oxa‐1, 3‐diazol‐4‐yl) amino)‐2‐deoxyglucose (2‐NBDG) were purchased from Thermo Fisher Scientific (Waltham, MA, USA). Fetal bovine serum (FBS) and horse serum (HS) for cell culture were bought from Hyclone (Logan, UT, USA).

### 2.2 Miracle fruit source

Fresh miracle fruit berries were obtained from the Taiwan Agricultural Research Institute, COA, Executive Yuan (Taichung, Taiwan, ROC) and were stored at −80°C before extraction. The seeds were separated from the skin and pulp (termed skin–pulp hereafter). The skin–pulp (MF) and seed (MS) parts were then washed and air dried separately. The yield of the fresh seed was 34.75% (w/w), and that of the fresh skin–pulp was 62.02% (w/w).

### 2.3 Extraction method

After cleaning, the skin–pulp (MF) and seed (MS) were separately crushed in 95% ethanol in a blender before extraction. The samples were extracted three times by using 10% of ethanol (95%); the samples were mixed using vortex mixer at room temperature for 3 hr and then kept for overnight for 4°C. The extracted solution was filtered, and the residue was repeatedly extracted. The filtered ethanol portions were combined and then evaporator. The yield of MS was 10.52% (w/w), and that of MF was 10.53% (w/w). Both extracts were stored at −20°C for further analyses (Supporting Information Figure S1). The extracts were weighed; subsequently, for each extract, a stock solution (100 mg/ml) was prepared in phosphate‐buffered saline (PBS) containing 0.2% DMSO. The effects of the extracts on glucose uptake in the C2C12 cell model were analyzed using this stock solution (Supporting Information Figure S2).

### 2.4 Determination of polyphenol content of miracle fruit extracts

#### 2.4.1 Total phenolic assay

Total phenolic content was determined using the modified Folin–Ciocalteau phenol method (Julkunen‐Tiitto, 1985). In brief, 50 μl of an appropriate dilution of the extracts (1 mg/ml), 2 ml of double‐distilled water, and 1 ml of the Folin–Ciocalteau reagent were added and vortexed for 1 min at room temperature; the mixture was then neutralized with 2.5 ml of 20% sodium carbonate (w/v). After 20 min of incubation at room temperature, the absorbance of the samples and standard solutions was measured at 735 nm on a spectrophotometer (Biotek, VT, USA). Gallic acid (0, 0.2, 0.4, 0.6, 0.8, and 1 mg/ml) was used to plot a standard curve. Total phenolic content is expressed as milligram gallic acid equivalents (GAE) per gram of the MF or MS extract.

#### 2.4.2 Total flavonoid assay

The total flavonoid content of the MF and MS extracts was estimated using the modified aluminum chloride colorimetric method (Chang, Yang, Wen, & Chern, 2002). In brief, ethanol extracts (1 mg/ml) were mixed with 5% aluminum chloride (2:1 (v/v)). After 30 min of incubation at room temperature, absorbance was measured at 415 nm on the spectrophotometer. Quercetin was used to plot a standard curve. Total flavonoid content of the extracts is expressed as milligram quercetin equivalents per gram of ethanol extracts (mg QE/g).

#### 2.4.3 Condensed tannin assay

Condensed tannin content was determined using the widely applied vanillin–HCl method (Broadhurst & Jones, 1978). In brief, 100 μl of the sample (1 mg/ml) was added to 1 ml of vanillin reagent (4% (w/v) in methanol) and 500 μl of concentrated HCl. After 20 min of incubation at room temperature, absorbance was used to plot a standard curve. Condensed tannin content is expressed as milligram (+)‐catechin equivalents per gram (mg CE/g) of ethanol extract. All colorimetric assays were performed in triplicate.

### 2.5 Determination of antioxidant capacity of miracle fruit extracts

#### 2.5.1 Trolox equivalent antioxidant capacity

Trolox equivalent antioxidant capacity (TEAC) denotes the antioxidant capacity of the compound under study to scavenge the blue‐colored ABTS^•+^ (2, 2′‐azino‐bis (3‐ethylbenzthiazoline‐6‐sulfonic acid) radical cation relative to the scavenging activity of the water‐soluble vitamin E analog, namely Trolox (Miller, Rice‐Evans, & Davies, 1993). Final concentration of 10 μM ABTS, 50 μM hydrogen peroxide, and 4.4 unit/ml peroxidase were mixed to prepare the ABTS^•+^ working solution, which was stored in the dark at 30°C for 1 hr. ABTS^•+^ formation was determined by measuring absorbance at 734 nm. For measuring antioxidant capacity, 30 μl of the sample (1 mg/ml) was mixed with 970 μl of the radical solution, and absorbance at 734 nm was measured within 1 min of mixing. Trolox was used as a positive control and was used to a plot a standard curve. The results are expressed as milligram Trolox equivalents per gram (mg Trolox eq. /g) of ethanol extract.

#### 2.5.2 DPPH (α, α‐Diphenyl‐β‐picrylhydrazyl) free radical scavenging activity

DPPH scavenging activity was measured using the method of Shimada, Fujikawa, Yahara, and Nakamura (1992) with slight modification. In brief, 500 μl of the MF or MS sample (1, 0.5, 0.25, 0.125, and 0.0625 mg/ml) was mixed with 1,000 μl of 0.5 mM ethanolic DPPH solution in a dark Eppendorf tube and incubated in a 37°C water bath for 30 min. Trolox (0.250, 0.125, and 0.0625 mg/ml) was used as a positive control. Ethanol was used as a blank control. Moreover, 200 μl of the reaction solution was added to a 96‐well plate, and absorbance was measured at 517 nm. The inhibitory percentage of the DPPH radical (DPPH radical scavenging activity (%)) was calculated using the following equation:

$$\begin{array}{cc} & \text{DPPH radical scavenging activity}\;(\%)=\\ & [1-\left(A_{\mathrm{sample}}\;\text{at}\;517\;\text{nm}/A_{\mathrm{control}}\;\text{at}\;517\;\text{nm}\right)]\;\times \;100 \end{array}$$

The amounts of sample to reduce the absorbance of DPPH by 50% (IC50) were calculated.

#### 2.5.3 Ferrous ion chelating activity

The ferrous ion chelating activity of the MF and MS extracts was estimated using the method of Dinis, Madeira, and Almeida (1994) with slight modification. In brief, 10 μl of different concentrations of the MF or MS sample (1, 5, 10, and 20 mg/ml), 1,000 μl of methanol, and 30 μl of 2 mM iron (II) chloride tetrahydrate were mixed for 30 s. Subsequently, 60 μl of 5 mM ferrozine was added, and the mixture was incubated at room temperature for 10 min. The absorbance of the ferrous iron–ferrozine complex was measured at 560 nm on the spectrophotometer. The ferrous ion chelating activity of the extracts was calculated using the following equation:

$$\begin{array}{cc} & \text{Ferrous ions}\;\left({\text{Fe}}^{2+}\right)\;\text{chelating activity}\;\left(\%\right)=\\ & [1-\left(A_{\mathrm{sample}}\;\text{at}\;560\;\text{nm}/A_{\mathrm{control}}\;\text{at}\;560\;\text{nm}\right)]\;\times \;100 \end{array}$$

### 2.6 Cell model

#### 2.6.1 C2C12 myoblast cell culture

The C2C12 mouse myoblasts cell line (BCRC number 60083) was purchased from the Food Industry Research and Development Institute (Hsinchu, Taiwan, ROC). The C2C12 cell was maintained in the high‐glucose Dulbecco's Modified Eagle Medium (DMEM; 4.5 g glucose/L, Invitrogen Co., CA, USA) supplemented with 10% FBS, 1.5 g/L sodium bicarbonate, and 1% penicillin–streptomycin. C2C12 cells were maintained in this medium at 37°C in a humidified atmosphere with 5% CO_2_.

#### 2.6.2 Differentiation of C2C12 to myotube cells

For differentiation to myotubes, C2C12 cells were reseeded in 6‐well plates for the creatinine kinase activity assay, a 96‐well plate for the MTT and 2‐NBDG glucose uptake assays, and a 10‐cm dish for the enzyme‐linked immunosorbent assay (ELISA) at a density of 1 × 10^5^ cells/ml (Supporting Information Figure S2) and were allowed to attach for 24 hr. After the cells reached 80%–90% confluence, the medium was switched to the differentiation medium (i.e., DMEM containing 2% (v/v) HS), and the medium was replaced every other day until myotubes formation was achieved after 6 days of incubation (Veliça & Bunce, 2011).

#### 2.6.3 Creatine kinase activity assay

To analyze the correlation between the time (days) and degree of differentiation to myotubes, the creatine kinase activity assay was performed using a commercially available calorimetric assay kit (BioVision, CA, USA). Cellular creatine levels were quantified from a standard creatine curve. Creatine levels are expressed in mU/ml and were normalized to the amount of protein (mg of total protein) by using a standard protein assay. Moreover, creatine levels are presented as a creatine:protein ratio (mU/g). The protein concentration of each sample was determined using the Bradford method (Bradford, 1976).

### 2.7 Glucose uptake assay

#### 2.7.1 Experimental design

After differentiation to myotubes, C2C12 cells were divided into the following groups: control, MF, MS, and positive control groups. In the control group, C2C12 myotube cells were cultured in glucose‐free, serum‐free DMEM only. In the MF group, the cells were cultured with the MF extract (final concentrations of 0, 1, 5, 10, 50, 100, 500, and 1,000 μg/ml) in glucose‐free, serum‐free DMEM. In the MS group, the cells were cultured with the MS extract (final concentrations of 0, 1, 5, 10, 50, 100, 500, and 1,000 μg/ml) in glucose‐free, serum‐free DMEM. In the positive control group, the cells were cultured with metformin (a final concentration of 1 mM) in glucose‐free, serum‐free DMEM.

#### 2.7.2 Measurement of C2C12 cell viability

Cell viability was measured using the MTT assay (Mosmann, 1983). The differentiated cells were washed with PBS and were then cultured with the MF or MS extract or metformin in glucose‐free, serum‐free DMEM for 0, 4, 24, and 48 hr. Briefly, 100 μl of MTT working solution (0.5 mg/ml MTT in PBS) was added to each well and incubated for 3 hr at 37°C. After the MTT solution was removed, the purple formazan crystals formed inside the cells were dissolved by adding 200 μl of DMSO to each well. Absorbance was measured at 570 nm. The cell viability of the control group at 0 hr was set to represent 100% cell viability. Cell viability was calculated using the following equation:

$$\text{Cell viability}\;\left({\%}_{\mathrm{control}}\right)=\left(A_{\mathrm{sample}}\;\text{at}\;570\;\text{nm}/A_{\mathrm{control}}\;\text{at}\;570\;\text{nm}\right)\;\times \;100$$

#### 2.7.3 2‐NBDG glucose uptake assay

For the glucose uptake assay, differentiated C2C12 cells were incubated with the fluorescent D‐glucose analog 2‐NBDG, which was used as a tracer to monitor glucose uptake in the cells (Zou, Wang, & Shen, 2005). The cells seeded in 96‐well plates were washed with PBS, incubated with or without insulin (150 nM), and subjected to the control, positive control, (metformin), or treatment conditions (MS or MF extract). Subsequently, the cells were incubated with 2‐NDBG (a final concentration of 100 mM) for an additional 2 hr. After treatment, free 2‐NBDG was washed out using PBS, and 200 μl of DMSO was added to each well to lyse the cell membrane. The fluorescence intensities of cells were measured at an excitation wavelength of 485 nm and an emission wavelength of 538 nm on using a fluorescence microplate reader (Molecular Devices, CA, USA). The glucose uptake of the control group without insulin treatment was set to represent 100% 2‐NBDG uptake. The 2‐NBDG uptake ratio was calculated using the following equation:

$$\begin{array}{cc} & \text{2‐NBDG uptake ratio}\;({\%}_{\mathrm{control}})=\\ & (A_{\mathrm{sample}}\;\text{at}\;485/538\;\text{nm}/A_{\mathrm{control}}\;\text{at}\;485/538\;\text{nm})\;\times \;100 \end{array}$$

### 2.8 ELISA process

#### 2.8.1 Cell lysate preparation

For ELISA, C2C12 cells were reseeded in a 10‐cm petri dish at a density of 1 × 10^5^ cells/ml. After differentiation, the cells were subjected to the control, positive control, (metformin), or treatment conditions (MS or MF extract) and were incubated with or without insulin for 2 hr. Subsequently, the cells were washed with PBS and were lysed using lytic buffer solution (Millipore, MA, USA) mixed with the protease inhibitor (Thermo, MA, USA). Then, the cell lysate was collected by centrifuged at 1,200 *g* for 15 min at 4°C and stored at −20°C for further analysis.

#### 2.8.2 ELISA analysis

All cell lysates were analyzed using the mouse IR ELISA kit, mouse GLUT4 ELISA kit, and Phosphoseek^™^ PI3K assay kit in triplicates. All three commercial ELISA kits were performed according to the manufacturer's instructions and were based on the quantitative sandwich format. All results were normalized to the amount of protein (mg of total protein) determined using the Bradford method.

### 2.9 Statistical analysis

All data are expressed as mean ± standard deviations (*SD*), and all experiments were repeated in triplicate. Statistical analyses were performed using SPSS (version 19, Chicago, IL, USA). The significance of the difference among the groups was measured using analysis of variance (ANOVA) with Tukey's multiple comparison adjustment. The Student *t* test was used to compare the difference in 2‐NBDG glucose uptake and ELISA analysis results among the groups with or without insulin treatment and was used to compare the difference in phenolic and antioxidant assay results between the MF and MS groups. *p *<* *0.05 was considered statistically significant.

## 3 RESULTS AND DISCUSSION

### 3.1 Total phenolic, total flavonoid, and condensed tannin contents of miracle fruit extracts

The results for the total phenolic, total flavonoid, and condensed tannin contents of the MF and MS extracts are summarized in Table 1. The total phenolic content of the MF extract (20.65 mg GAE/g) was almost two times higher than that of the MS extract (10.51 mg GAE/g). Inglett and Chen (2011) also demonstrated that miracle fruit pulp extracts (22.58 mg/g) showed significantly higher total phenolic contents than MS extracts (18.55 mg/g). A similar trend was also observed for total flavonoid content. The total flavonoid content of the MF extract (3.3 mg QE/g) was approximately 10 times higher than that of the MS extract (0.3 mg QE/g). This study also showed the condensed tannin content of the MS extract was significantly higher (30.83 mg CE/g). Both total phenolic and total flavonoids contents have been used as important indicators of antioxidant capacity (Rice‐Evans, Miller, & Paganga, 1996). These results provided strong evidence of the high antioxidant capacity of miracle fruits.

**Table 1:** Phenolic content and antioxidant capacity in each gram of MF and MS^a^

Group	Phenolic contents			Antioxidant capacity		
	Total phenolic (mg GAE/g)	Total flavonoids (mg QE/g)	Condensed tannin (mg CE/g)	TEAC (mg trolox eq./g)	DPPH radical scavenging, IC50 (mg/ml)	Ferrous ions chelating activity, IC50 (mg/ml)
MF	20.65 ± 0.10^a^	3.3 ± 0.33^a^	7.50 ± 0.00^b^	31.51 ± 0.11^a^	0.90 ± 0.06^b^	46.67 ± 13.07
MS	10.51 ± 0.12^b^	0.3 ± 0.00^b^	30.83 ± 0.00^a^	12.00 ± 0.40^b^	6.31 ± 0.41^a^	61.87 ± 08.07

*Notes*

Data with different superscript letters in the same column denote a significant difference (*p *<* *0.05).

a Results are expressed as mean ± *SD*.

### 3.2 Antioxidant capacity

Many studies have shown that the potential antioxidant capacity of polyphenols can be attributed to their ability to donate hydrogen atoms (Goupy, Dufour, Michele Loonis, & Dangles, 2003), scavenge radicals (Re et al., 1999), and interrupt oxidation reaction chains or chelate metals (Morel et al., 1993). However, the mechanism of the antioxidant capacity of miracle fruit remains unclear. The present study determined the phenolic content and antioxidant capacity of MF and MS extracts, as shown in Table 1. The TEAC value and DPPH radical scavenging activity of the MF extract (TEAC: 31.51 mg Trolox eq./g; DPPH: 52.60%) were both significantly higher than those of the MS extract (TEAC: 12.00 mg Trolox eq./g; DPPH: 10.87%). The high antioxidant capacity of miracle fruit may be related to the high total phenolic and flavonoid contents. Similarly, a previous study found high polyphenol content in grape skin extracts presenting high antioxidant capacity (Molina‐Quijada, Medina‐Juárez, González‐Aguilar, Robles‐Sánchez, & Gámez‐Meza, 2010). The DPPH assay is a rapid method for determining antioxidant capacity; it measures the ability of the compound under study to donate hydrogen atoms to the DPPH radical, resulting in the scavenging of the DPPH radical. Antioxidant capacity was evaluated by calculating IC50 values, representing the concentration at which 50% of radical scavenging activity is inhibited. The IC50 values are listed in Table 1. Higher scavenging activity implies higher antioxidant capacity, which reflects lower IC50 values (Brand‐Williams, Cuvelier, & Berset, 1995). The MF extract at 1 g/ml showed the highest radical scavenging activity (52.60%, *p *<* *0.05), which was much higher than that of the MS extract (10.87%). Compared with the positive control Trolox (IC50 = 0.18 mg/ml), the order of IC50 values determined from the DPPH radical scavenging assay was Trolox < MF (0.9 mg/ml) < MS (6.3 mg/ml). Both the MF and MS extracts showed excellent DPPH radical scavenging activity, with IC50 values <50 mg/ml. The data also showed a linear correlation between MF or MS concentrations and the inhibitory percentage of DPPH (data shown in Supporting Information Figure S3a). Katalinic, Milos, Kulisic, and Jukic (2006) demonstrated a linear correlation between total phenolic content and antioxidant capacity. In the present study, no significant difference was observed in FRAP assay results between 1 mg/ml MF and 1 mg/ml MS extracts. However, the data also showed a linear correlation between MF and MS concentrations and the percentage of ferrous ion chelating capacity (data shown in Supporting Information Figure S3b). According to the FRAP assay, the ferrous ion chelating capacity of the 20 mg/ml MF extract was significantly higher from that of the 20 mg/ml MS extract (data not shown).

### 3.3 Cell model

Skeletal muscle plays a crucial role in balancing energy metabolism, particularly glucose homeostasis (Kelley et al., 1988). To investigate the level of C2C12 cell differentiation, the creatine kinase activity in the cell lysate was measured on days 0, 2, 4, 6, 8, 10, and 12 of cell differentiation. The highest creatine kinase activity was shown on day 6 of cell differentiation (Supporting Information Figure S4). Thus, within 6 days, C2C12 myoblasts incubated in 2% HS completely differentiated into myotube cells (Supporting Information Figure S4a).

In the MTT assay (Supporting Information Figure S5), regardless of whether the MF or MS extract was used, miracle fruit extracts at concentrations ranging from 1 to 500 μg/ml showed no cytotoxicity to cells incubated with the extracts for 24 hr (cell viability was higher than 75%). In previous reports, 1 mM metformin showed no cytotoxicity to differentiated C2C12 myotubes (Park et al., 2014). Our data also indicated that in cells incubated with metformin for 48 hr, 1 mM metformin showed no cytotoxicity (Supporting Information Figure S5). Furthermore, the data showed negative correlation between the concentration of MS and cell viability; after treatment with the highest concentration of MS (1,000 μg/ml; Supporting Information Figure S5b), cell viability suddenly decreased to <50% in 4 hr. Therefore, concentrations ranging from 1 to 500 μg/ml for the MF and MS extracts and the concentration of 1 mM for metformin were used for additional cell experiments.

**Figure 1:**
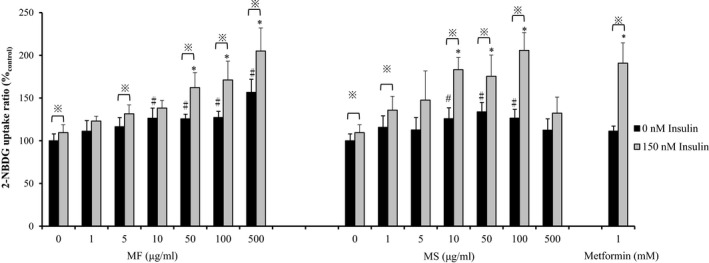
Effects of miracle fruit extracts (MF and MS) on 2‐NBDG uptake in the presence or absence of insulin. The results are expressed as mean ± *SD*. To determine significant differences (*p *<* *0.05), data were analyzed using SPSS through ANOVA with Tukey's multiple comparison adjustment. #Samples versus control (0 μg/ml) without insulin (0 nM insulin). *Samples versus control (0 μg/ml) with insulin treatment (150 nM insulin). ※The results were analyzed using the Student *t* test to compare the difference between samples with insulin treatment versus those without insulin treatment

As shown in Figure 1, in control cells, 150 nM insulin predominantly increased glucose uptake to approximately 9.53% (*p *<* *0.05). Furthermore, in cells with insulin treatment, metformin also increased glucose uptake to approximately 79.54% (*p *<* *0.05). Compared with control cells, miracle fruit extracts induced glucose uptake in cells with or without insulin treatment. The MF extract (10–500 μg/ml) increased glucose uptake to approximately 25.7%–56.6% (*p *<* *0.05), which was higher than that induced by the MS extract (10–100 μg/ml). The MS extract (10–100 μg/ml) also significantly increased 2‐NBDG uptake to approximately 25.9%–33.8% in cells without insulin treatment. In C2C12 cells with insulin treatment, the MF extract (50–500 μg/ml) significantly increased glucose uptake to 162.2%–205.0%, and the MS extract (10–100 μg/ml) increased glucose uptake to 183.3%–205.3% (*p *<* *0.05). The MS extract at 500 μg/ml could not effectively enhance 2‐NBDG uptake. This finding may be because of the higher cytotoxicity of the 500 μg/ml MS extract. Compared with the positive control metformin, the MF extract at 500 μg/ml showed higher efficacy for glucose uptake in cells treated with or without insulin.

### 3.4 ELISA

Two major signaling pathways regulate glucose metabolism in skeletal muscle: the insulin signaling pathway and the 5′ adenosine monophosphate‐activated protein kinase pathway (Khan & Pessin, 2002). To investigate the effect of miracle fruit extracts (MF and MS) on the insulin signaling pathway, the expression of IR‐PI3K/GLUT4 in C2C12 cells was examined using a commercial ELISA kit. In the present study, metformin was used as a positive control; metformin can enhance the insulin signaling pathway in skeletal muscle by increasing the tyrosine phosphorylation of IR and PI3K (Kumar & Dey, 2002). Metformin also increases GLUT4 expression in myoblasts (Sarabia, Lam, Burdett, Leiter, & Klip, 1992).

**Figure 2:**
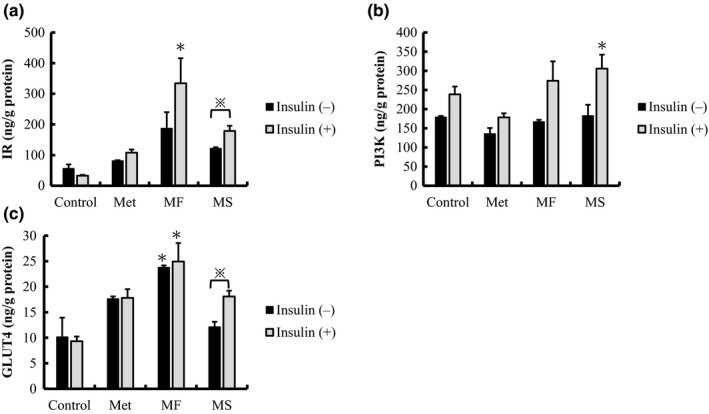
ELISA analysis of effects of miracle fruit extracts on insulin‐stimulated protein expression in C2C12 cells. (a) Insulin receptor (IR), (b) phosphatidylinositol 3‐kinase (PI3K), and (c) glucose transporter 4 (GLUT 4) expression in insulin‐stimulated cells when treated with 1 μg/ml of the MF or MS extract. All data are shown as mean ± *SD* in triplicate. To determine significant differences, data were analyzed using SPSS through ANOVA with Tukey's multiple comparison adjustment (*p *<* *0.05). *Samples versus control (0 μg/ml) without insulin treatment (0 nM insulin). ※The results were analyzed using the Student *t* test to compare the difference between samples with insulin treatment versus those without insulin treatment

As illustrated in Figure 2, compared with control cells, the MF extract increased the expression levels of IR and GLUT4 by 2.3 and 3.3 times, respectively, in cells without insulin treatment. However, in cells with insulin treatment, the MF extract significantly increased the expression levels of IR and GLUT4 by 5.9 and 2.5 times, respectively (*p *<* *0.05). The MS extract also increased the expression levels of IR‐PI3K/GLUT4. Nevertheless, the expression levels in the MS group were lower than those in the MF group, although the difference was not significant. However, in cells with insulin treatment, the MS extract significantly increased PI3K expression by 1.7 times (*p *<* *0.05). Similarly, the MF extract significantly increased PI3K expression by 1.5 times. Compared with metformin, the MF extract increased the expression levels of IR and GLUT4 in cells with insulin treatment. This study clearly revealed that miracle fruit ethanolic extracts, particularly MF, exerted antidiabetic effects through the activation of the insulin signaling pathway by increasing IR‐PI3K and GLUT4 expression in C2C12 skeletal muscle cells.

## 4 CONCLUSIONS

Our study revealed that miracle fruit extracts, particularly MF, exhibited high phenolic content and antioxidant capacity. The positive correlation between total phenolic and antioxidant activities has been well established. According to 2‐NBDG uptake test, both MF and MS can effectively increase the glucose uptake; however, high dose of MS extract showed higher cytotoxicity; hence, we suggest that MF might be a safer material for future food application. The results of analysis indicate that the miracle fruit ethanolic extracts, particularly MF, increased glucose uptake through the activation of the insulin signaling pathway by increasing IR‐PI3K and GLUT4 expression. Based on these results, miracle fruit extracts can improve type 2 DM through the activation of the insulin signaling pathway and antiglycation activity. In a future study, we will focus on the factors contributing to the glucose regulation of functional phytochemicals in miracle fruit and their underlying mechanism.

## CONFLICT OF INTEREST

All authors declare no conflicts of interest, and there is no funding to report for this submission.

## ETHICAL STATEMENT

The current study does not involve any human or animal testing, hence not required to complete an ethical assessment.
