# Supplementary material for: Modulatory effects of miracle fruit ethanolic extracts on glucose uptake through the insulin signaling pathway in C2C12 mouse myotubes cells
Source: Food Sci Nutr. 2019 Feb 5;7(3):1035–42. doi: 10.1002/fsn3.935 (PMC6418466; doi:10.1002/fsn3.935)
Supplement: Supplementary file 1 [file FSN3-7-1035-s001.docx]

**Supplementary data**

Miracle fruit

Washing, blending

95% Ethanol

Filtrate

Rotary evaporator

Flesh (MF)

Seed (MS)

Polyphenolic analysis

1. Total phenols
2. Total flavonoids
3. Condensed tannin


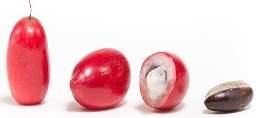

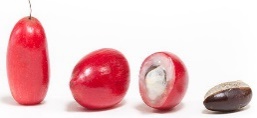

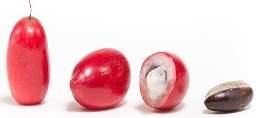


Antioxidant analysis

1. TEAC
2. DPPH
3. FRAP

Anti-glycation assay

1. BSA / Fructose
2. BSA / MGO

Fig. S1. Experimental design. Sample extraction and functional assessment.

Fig. S2. Experimental design. Diabetes cell model establishment and glucose uptake assay.

C2C12 cells

Myotube

Control

(PBS replacement)

1. IR
2. GLUT4
3. PI3K

Differentiation

1. Morphology
2. Creatine kinase

Diabetes model

(Insulin (+/−) )

MTT assay

(0, 24, 48 hours)

Positive control

(1 mM Metformin)

MF

MS

Cytotoxicity testing

Glucose uptake assay

2-NBDG uptake

Insulin signaling pathway

(ELISA)

1,5,10,50,100,500,1000 μg/mL


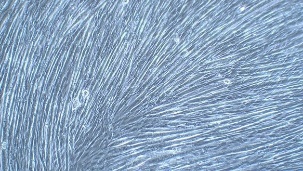

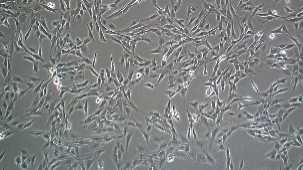


S3a.

S3b.

Fig. S3. Linear relationship between antioxidant capacity and concentration of MF and MS extracts^1^. S3a. DPPH assay^2^; S3b. FRAP assay.

^1^The formula beside the correlation line in the figure expresses the correlation between the sample concentration and the scavenging ability of DPPH or FRAP.

^2^The standard agent was Trolox (250, 125, and 62.5 µg/mL). The gray line shows IC50, which is the DPPH radical scavenging ability (%) at 50%.


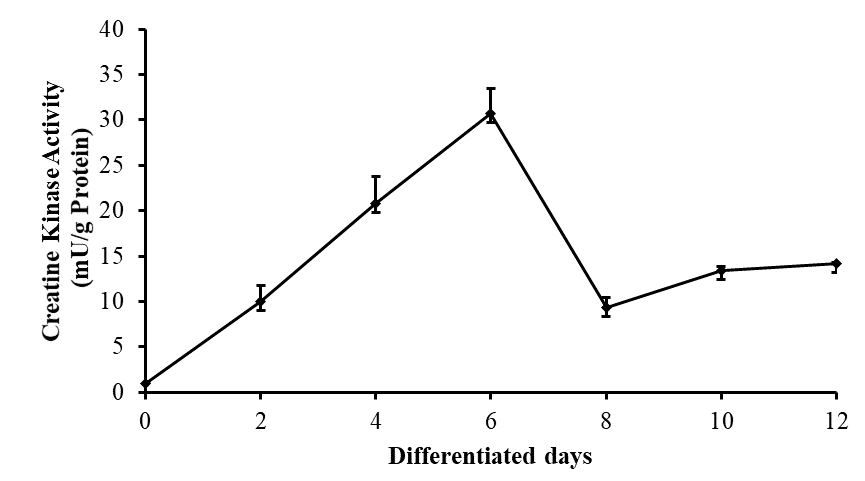

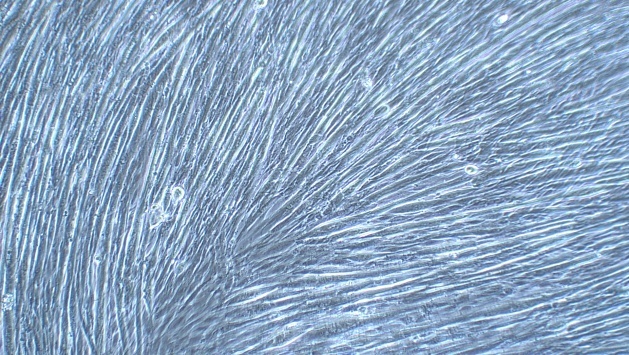


S4a.

Fig. S4. Creatinine kinase kinase activity on various days (0, 2, 4, 6, 8, 10, and 12 days) of differentiation of C2C12 cells^1^.

S4a. Cell morphology of differentiated C2C12 myotube cells.

^1^Results are expressed as mean ± SD. The creatine kinase activity represents the level of differentiated C2C12 cells.

S5a.

S5b.

Fig. S5. Effects of miracle extracts on viability of C2C12 cells.

Cell viability ratio (%) = OD_570 nm_ of sample/OD_570 nm_ of control_0 time point_ ×100.

Results are expressed as means at different time points (0, 4, 24, and 48 hours). S5a. Cell viability in the MF group. S5b. Cell viability in the MS group.

**IR**

**Insulin**

**Glucose**

**MF**

**MS**

**GLUT4**

**>**

AGER

**AGEs**

Fig. S6. Proposed effects of MF and MS extracts on the interaction between the insulin signaling pathway and AGE formation.

AGE formation was downregulated by MF and MS extracts. MF and MS groups upregulated the expression of IRPI3K and GLUT4 and then increased the glucose uptake ability of muscle cells.
